# Engineering Organoid Vascularization

**DOI:** 10.3389/fbioe.2019.00039

**Published:** 2019-03-19

**Authors:** Sergei Grebenyuk, Adrian Ranga

**Affiliations:** Laboratory of Bioengineering and Morphogenesis, Department of Mechanical Engineering, KU Leuven, Leuven, Belgium

**Keywords:** organoid, vascularization, bioengineering, biofabrication, biomaterials

## Abstract

The development of increasingly biomimetic human tissue analogs has been a long-standing goal in two important biomedical applications: drug discovery and regenerative medicine. In seeking to understand the safety and effectiveness of newly developed pharmacological therapies and replacement tissues for severely injured non-regenerating tissues and organs, there remains a tremendous unmet need in generating tissues with both functional complexity and scale. Over the last decade, the advent of organoids has demonstrated that cells have the ability to reorganize into complex tissue-specific structures given minimal inductive factors. However, a major limitation in achieving truly *in vivo*-like functionality has been the lack of structured organization and reasonable tissue size. *In vivo*, developing tissues are interpenetrated by and interact with a complex network of vasculature which allows not only oxygen, nutrient and waste exchange, but also provide for inductive biochemical exchange and a structural template for growth. Conversely, *in vitro*, this aspect of organoid development has remained largely missing, suggesting that these may be the critical cues required for large-scale and more reproducible tissue organization. Here, we review recent technical progress in generating *in vitro* vasculature, and seek to provide a framework for understanding how such technologies, together with theoretical and developmentally inspired insights, can be harnessed to enhance next generation organoid development.

## *In vitro* Self-Organization: Toward Tissue-Level Complexity

Organoids have been described as potentially transformational new model systems, which could create dramatic efficiencies in the drug discovery process and could become the building blocks for large-scale engineered tissues. Indeed, organoids bridge the gap between readily accessible and easily scalable traditional 2D *in vitro* cell culture and the complexity of animal models. Derived from pluripotent stem cells or self-renewing tissue progenitor stem cells, organoids have been generated for an increasing variety of organs, including intestine, kidney, brain, retina, liver and spinal cord. The self-organization process of organoids is uniquely manifested in three-dimensional culture systems, which resemble, to some extent, the *in vivo* organ or tissue from which they were derived, and which thereby allow for biologically relevant cell-cell and cell-matrix interactions (Clevers, 2016). In contrast to 2D cell culture, organoids provide a more realistic physiological response as they bear more physical and molecular similarity to their tissue of origin. Indeed, it has been observed that the signaling pathways governing organoid formation are similar to those utilized during *in vivo* organ development and homeostasis (Hynds and Giangreco, 2013; Camp et al., 2015). Another unique aspect of organoid models is the possibility to genetically manipulate human tissue, thereby opening unprecedented therapeutic perspectives for diseases characterized by genetic abnormalities. Indeed, the aberrant physiological effects of genetic disorders can be modeled by generating organoids from patient-derived induced pluripotent stem cells or by introducing known disease-specific mutations. Personalized medicine and cost-effective development of therapies for rare diseases is a recent example of the ongoing evolution of the field (Dekkers et al., 2013, 2016). Organoids are therefore extremely relevant to model human biology *in vitro*, and, as a consequence, are becoming cost-effective ways to develop therapies for human disease. The use of organoids is now increasingly being translated in areas of personalized medicine and drug discovery (Ranga et al., 2014a; Kelava and Lancaster, 2016), with initiatives already under way to turn organoid-based models into scalable and practical tools, including through institutional efforts to establish data banks of human cancer models in the form of organoids. For example, the molecular repertoire of gastric cancer organoids derived from 34 patients was recently characterized, and a subset of these were treated with a library of anti-cancer drugs, which enabled both the identification of effective new compounds for gastric cancer, as well as the demonstration of the possibility of repurposing a drug previously approved for breast cancer (Yan et al., 2018), all on the basis of specific effectiveness on molecular sub-categories of the disease. In addition to the three-dimensional cellular organization which is considered more relevant at the level of cellular identity and gene expression, the unique functional readouts emerging in organoid model systems are also beginning to be exploited for diagnostic applications. One striking recent example is in the establishment of forskolin-induced intestinal organoid swelling as a biomarker of cystic fibrosis transmembrane conductance regulator (CFTR) function (de Winter-de Groot et al., 2018). This novel functional *in vitro* assay was used to stratify patients and to tailor drug cocktails to individual patients based on the *in vitro* response of their organoid (Dekkers et al., 2016).

Although organoids have gained significant attention in the academic community and have now achieved increasing exposure in the pharmaceutical industry, there remain major challenges that prevent such models from achieving a broader deployment. One of the key limitations in using organoid-based approaches to generate functional tissue is that upon reaching a certain size, organoids cease to proliferate and develop a necrotic core. The process of growth arrest is thought to be linked to two phenomena: a switch from a proliferative, stem-like state to a non-proliferative, terminally differentiated one, as well as the loss of cell viability in the inner core of the organoid and subsequent necrosis upon reaching a limiting size beyond which diffusion alone can no longer allow for oxygen, nutrient and metabolite exchange. The rapid induction of terminal differentiation or aggregation of defined number of pre-differentiated cells are strategies used to limit the size of organoids, which are then more commonly referred to as spheroids. In order to maintain the complexity and scale of organoids it is therefore necessary to prevent the appearance of the necrotic inner core leading to the premature differentiation in the outer layers of the organoid. This phenomenon, in turn, can largely be ascribed to a lack of organoid vascularization and achieving such vascularization of organoids therefore remains a major challenge in the field. Strategies for enabling organoid vascularization can be analogous to the long-standing goal of generating vascularised tissue in the context of tissue engineering, however fundamental biological differences between engineered bulk tissues and complex organoid organization must be taken into account. Indeed, it long been realized that, with the exception of a few avascular tissues, any attempts at generating larger scale tissue constructs would be limited to a length scale of approximately 150 μm imposed by the natural diffusion limit of oxygen and nutrients in tissue. Recent reviews (Miller et al., 2012; Kim et al., 2016; Kinstlinger and Miller, 2016) have highlighted methods to overcome these limitations, which include approaches rooted in conventional microfluidics but which have also more recently evolved to include a variety of free-form 3D fabrication techniques. These include layer-by-layer deposition of materials, known as additive manufacturing, or selective removal of materials to form tubular voids connected to perfusion networks, as well as a range of hybrid approaches utilizing sacrificial materials. Here, we review the diversity of these approaches, focusing on techniques which have been proposed to recreate vasculature at various scales, and explore the potential and limitations of these platforms in interfacing with organoids.

## Bioprinting

**Figure 1:**
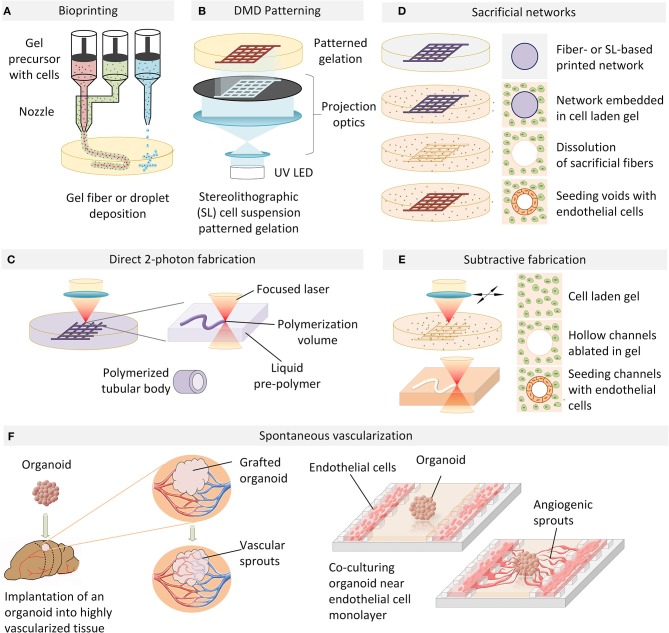
An overview of vascularization techniques. **(A)** Vascular structures are bioprinted by extrusion or droplet deposition of cells suspended in biocompatible gel in a patterned manner. **(B)** Photo-induced gelation of cell-containing liquid precursors is performed in a layer-by-layer fashion with DMD patterning. **(C)** 2-photon photopolymerization is used to directly fabricate perfusable tubular networks of arbitrary geometry which can be embedded in a cell-containing hydrogel. **(D)** A cell-laden extracellular matrix is cast over a sacrificial filament lattice created by bioprinting or stereo-lithography. In an aqueous environment, the sacrificial filaments are dissolved and the resulting hollow perfusable network is perfused with endothelial cells which form a conformal layer around the inner diameter of the vessels. **(E)** Microchannels fabricated in a cell-laden hydrogel by laser ablation can be directly seeded with endothelial cells to generate functional blood vessels. **(F)** Organoid angiogenesis can be achieved by grafting in a highly vascularized animal tissue, with the host vasculature infiltrating the organoid. Alternatively, organoid vascularization is also achieved *in vitro* by endothelial cell co-culture in compartmentalized microfluidic chip. VEGF and hypoxia gradients established on-chip provide spatial guidance cues to direct angiogenic sprouting.

**Figure 2:**
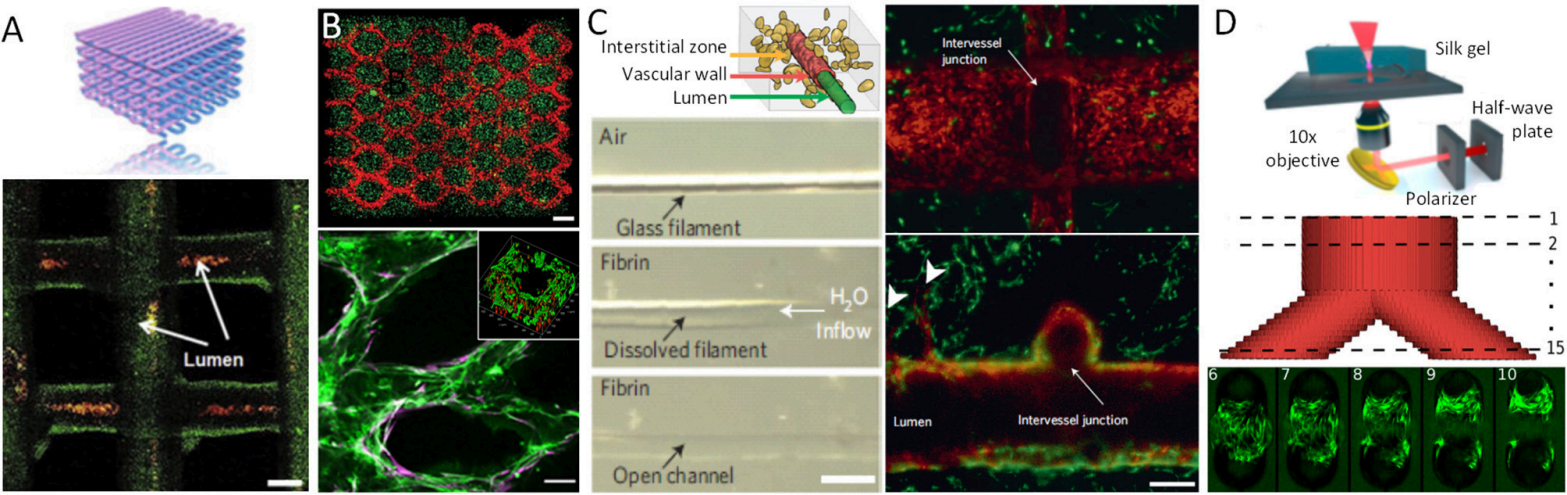
Examples of vascularized constructs and tissues. **(A)** Schematic vascular network formed by bio-ink extrusion and confocal image of the bio-printed tubular constructs containing green fluorescent beads, perfused with red fluorescent micro-beads inside the lumens. **(B)** DMD-based bio-printing of cell-laden tissue constructs with HUVECs (red) encapsulated in the intended channels and HepG2 (green) cells encapsulated in the surrounding area (top). Scale bars, 250 μm. HUVECs (Green, CD31) and supportive 10T1/2 mesenchymal cells (Purple, α-SMA) aligned within the patterned channel regions after 1-week *in vitro* culture (bottom). Insert indicates cross-section of the channel, demonstrating a lumen formed by biodegradation of the gel. Scale bars: 100 μm. **(C)** Schematic of the vascular lumen, endothelial cells lining the vascular wall, and the interstitial zone containing matrix and encapsulated cells (top). A single sacrificial carbohydrate-glass fiber is encapsulated in a fibrin gel. After immersion in aqueous solution the fiber is dissolved (middle) yielding an open perfusable channel in the fibrin gel (bottom, scale bar, 500 μm). Representative vessel after 9 days in culture demonstrating endothelial monolayer (red) lining the vascular lumen, surrounded by 10T1/2 cells (green). Endothelial cells forming single and multicellular sprouts (arrowheads) from the patterned vasculature. Scale bars, 200 μm. **(D)** Multi-photon micromachining device generates hydrogel micro-channels allowing for fibroblast adhesion and growth within a Y-shaped feature 9 days after seeding. Scale bar, 100 μm.

Layer-by-layer deposition of hydrogels incorporating cells of interest has generally been explored in studies aiming at generating large-scale tissue constructs, and has been most frequently performed by either filament deposition or droplet based approaches. Fused filament fabrication (FFF) or fused deposition modeling (FDM) involves the extrusion of a cell-containing gel in the form of a thin filament and its deposition in a pre-programmed, controlled manner to form a specific pattern (Figures 1A, 2A). In the context of attempts to vascularise *in-vitro* tissue, FDM has been used to deposit endothelial cells (ECs) interleaved or combined with other cell types (Xu et al., 2012; Kinoshita et al., 2016; Roudsari et al., 2016; Zhang et al., 2016). Coaxial filaments have also been extruded, where the core of the filament incorporates endothelial cells while the external layer is composed of tissue-specific cells. As a proof-of-principle of this technique, functional adipose tissue pre-vascularized with an aligned endothelial vessel network was shown to anastomose with the host vasculature in a mouse model (Leong et al., 2013). A more versatile method consists in using composite materials which allow for sequential two-step crosslinking mechanisms. For example, a bioink consisting of gelatin methacryloyl (GelMA), sodium alginate, and 4-arm poly(-ethylene glycol)-tetra-acrylate (PEGTA) could first be ionically cross-linked by calcium ions to provide temporary structural stability during the bioprinting process, followed by covalent photo-crosslinking of GelMA and PEGTA to form stable constructs (Jia et al., 2016). Combining the use of composite materials with a multilayered coaxial extrusion approach is a strategy which allows for direct one-step bioprinting of larger scale constructs (Figure 1A). Gel extrusion methods, despite their relative versatility, do not reproduce the intertwined and highly branched network topology occurring *in vivo* due to their continuous mode of filament deposition. Another limitation of the approach is that the minimal diameter of vessels achievable is determined by the diameter of the nozzle used for extrusion: the smaller the diameter, the higher the required extrusion pressure and therefore the more detrimental to cells.

Some of these limitations can be overcome by depositing cells and matrices in a contactless manner in the form of droplets, thereby allowing for a more precise spatial control of the resulting pattern. Methods to generate and eject droplets from nozzles include local thermally induced vaporization (Cui et al., 2012), electrostatic (Xie et al., 2018) or piezoelectric (Saunders et al., 2008; Kim et al., 2010) droplet formation, or micro-valve controlled pulsed ejection (Dababneh and Ozbolat, 2014; Gudapati et al., 2016). Alginate is a typical material used in these applications: it is ejected into a CaCl_2_-containing solution which acts as both cross-linking agent and support material to provide buoyant force for the newly formed alginate gel. Well-defined vertical tubular structures with an inner diameter of ~200 μm (Nishiyama et al., 2008), as well as free-form fabrication of both horizontal and vertical vessel-like bifurcations has been demonstrated with this approach (Xu et al., 2014). Cell viability after bioprinting with most current methods, using alginate as well as a variety of other bioinks, is now well-established, with cell viabilities after printing on the order of 90% or more (Christensen et al., 2015). An additional advantage of these approaches is that a typical droplet has a volume in the picoliter range and contains only a few cells, thereby theoretically providing superior spatial resolution compared to filament-based deposition processes. In practice however, wetting and adhesion properties of the droplet-substrate interface significantly limit the effective achievable resolution (Nishiyama et al., 2008; Christensen et al., 2015). Conversely, discrete droplet based approaches are better suited to rapid and combinatorial switches in composition of several types of cells and matrices during the fabrication process, allowing for generation of cellular patterns of arbitrary complexity (Sala et al., 2011; Ranga et al., 2014b).

## Digital Micro-mirror (DMD) Patterning

Stereolithography-based additive manufacturing based on light-mediated cross-linking of photosensitized polymers (photopolymerization) has gained widespread adoption in medical and biological applications due to its primary advantage of enabling truly arbitrary 3D geometries with unrivaled resolution. In order to create pre-vascularized tissues with more complex architecture at higher resolution, approaches based on photopolymerization of cell-laden gel liquid precursors by digital micro-mirror (DMD) devices have been explored. Polymerization is selectively induced in regions exposed to illumination in a 2D pattern generated by the projection of an image bitmap via a micro-fabricated mirror array (Figure 1B). Complex layer-by-layer 3D structures can be formed by repetitive illumination of serial projected patterns, interleaved by cycles of washing out of the unexposed, unpolymerized liquid gel precursor. In an example of this approach, a complex network of gelatin-methacrylate gel with human umbilical vein endothelial cells (HUVEC) was first subjected to patterned UV illumination and washed with medium, followed by over-casting and polymerization of another gel containing hepatocellular HepG2 and 10T1/2 support cells (Zhu et al., 2017). All cell types localized to the designated sections, and endothelial cells were shown to form lumen-like structures spontaneously *in vitro*. When implanted under the dorsal skin of a severe combined immunodeficiency (SCID) mouse, the tissue constructs demonstrated progressive formation of an endothelial network and anastomosis with the host circulation. In contrast, patterned constructs without HUVEC cells did not demonstrate any vascularization upon implantation, suggesting that *in vitro* endothelial pre-vascularization may be a necessary requirement for successful engraftment *in vivo*. Intriguingly, while the initially DMD-formed HUVEC-laden channels had diameters of 100–150 μm, the resulting vessels were as large as 1 mm in diameter, and no functional microvasculature was evidenced. Significantly smaller branched channels with capillary structures of 25–45 μm have been demonstrated with the same technology in a simplified biomimetic honeycomb scaffold using PEG-diacrylate as a photo-crosslinkable hydrogel (Huang et al., 2014), suggesting the potential of projection-based stereolithography as a high-resolution technique for the free-form fabrication of capillary networks.

## Direct 2-Photon Fabrication

Two-photon stereo-lithography, based on non-linear two-photon absorption, provides selective photopolymerization of sub-micron volumes and allows for the generation of complex three-dimensional microstructures in a single processing step (Ovsianikov et al., 2012; Raimondi et al., 2012). In one of the earliest studies showing the potential of this approach, the fabrication of micro-compartments for bacterial colonies by *in situ* two-photon cross-linking of gelatin demonstrated the possibility of creating arbitrary spatial arrangements at the micrometer scale to allow interactions between defined cell populations (Connell et al., 2013). Being a particularly dimensionally flexible and accurate technique, two-photon stereolithography has been increasingly used in cell biology and bioengineering applications (Ovsianikov et al., 2011; Connell et al., 2013; Sun et al., 2015), including in recent attempts to directly fabricate perfusable networks (Figure 1C). In pioneering work on direct 2-photon fabrication of tubular structures, branching vessels with internal diameters under 20 μm could be achieved (Meyer et al., 2012) (Figure 2C). While this study did not demonstrate the perfusability of the generated networks, it paved the way for similar approaches based on the flexible and versatile design-on-demand capabilities of this technique. In one strategy making use of these 3D design capabilities, micro-pores were created in the walls of a branched network of relatively large (circa 1 mm) polyacrylate vessels, thereby enabling the successful lining of the main vessels with human dermal microvascular endothelial cells (Huber et al., 2016), as well as the migration of cells and medium exchange across the vessel walls at precisely defined spatial locations. While 2-photon-based photopolymerization methods appear to achieve unprecedented resolution, their limited throughput (Ovsianikov et al., 2011; Connell et al., 2013; Sun et al., 2015), limited choice of biocompatible photoiniators (Wu et al., 2011; Li et al., 2016), and considerable infrastructural cost have precluded their wider adoption.

## Sacrificial Networks

To create complex 3D networks, approaches based on removing, rather than adding material, have also been explored. Indeed a number of studies have been based on the idea that a sacrificial filament network can be embedded in a cell-containing matrix which, upon removal, leaves behind perfusable channels which can then be seeded with endothelial cells to form a vascular network with defined topology (Figure 1D). For example, a method for creating vascularized tissues which utilizes carbohydrate glass as a sacrificial material has been reported (Miller et al., 2012). An interconnected carbohydrate-glass lattice was printed (Figure 2D), encapsulated in ECM with live cells, and then dissolved within minutes in media without damage to nearby cells. In a similar approach, a sacrificial ink containing Pluronic and thrombin, and an cell-laden ink with gelatin and fibrinogen, was printed within a 3D perfusion chip (Kolesky et al., 2016). After printing, a matrix containing gelatin, fibrinogen, cells, thrombin, and transglutaminase was cast over the printed lattices. After thrombin-induced fibrinogen polymerization into fibrin and transglutaminase-induced cross-linking of the gelatin and fibrin, the fluidic chip was cooled and the sacrificial ink was solubilized and removed, leaving behind a vascular network coated with endothelial cells and connected to an external perfusion pump. Vascular networks obtained in this manner have been demonstrated to anastomose in a rat femoral artery graft model, with Doppler imaging confirming the vascular patency of the implants (Sooppan et al., 2016). A number of similar approaches involving sacrificial inks embedded in matrices have clearly established the possibility of creating thick vascularised tissue within perfusable fluidic chips with controlled composition and architecture, in some cases over several weeks (Golden and Tien, 2007; Wu et al., 2011; Bertassoni et al., 2014; Li et al., 2016). Droplet-based inkjet printing of gelatin has also been used to create sacrificial structures (Kolesky et al., 2016). However, the current limitations of most sacrificial network approaches have included the challenges of dimensional accuracy and precision, as well as of complete and homogenous removal of the sacrificial material, in particular for increasingly small and complex geometries. This may indeed account for the limits in achieved vessel dimensions of 150 μm (Miller et al., 2012), which are still an order of magnitude larger than the average capillary size.

In order to address some of these challenges, sacrificial perfusion networks have been generated using micro-stereolithographic processes with DMD-based instruments (Figure 2B). In one example, a custom DMD-based 3D printing device was used in a first step to polymerize a network of branching rods from a water-soluble photopolymer (Kang et al., 2016). The fabricated structure was coated with collagen and embedded in porous polycaprolactone (PCL) scaffold. The fabricated network was then dissolved in NaOH-containing solution and could be seeded with HUVECS, while the porous PCL scaffold was seeded with human lung fibroblasts to achieve a co-culture model system. This approach exemplifies what is expected to be an increasing trend toward deploying a mix of materials and fabrication technologies to achieve combinations of desired properties in complex co-culture platforms.

## Subtractive Fabrication by Laser Ablation

Existing microfabrication techniques utilizing stereolithography are not limited to photo-patterning of biochemical cues, direct 3D printing or direct laser writing of photopolymerizable materials. Indeed, elegant optical approaches for generating free-form channels *in situ* within cellularized hydrogel materials have also been demonstrated based on laser photo-ablation (Applegate et al., 2015) (Figures 1E, 2C). Induced by multiphoton absorption of light, photo-ablation allows for the generation of voids as small as 5 μm in diameter. Silk fibroin has been reported as an especially suitable material for this technique due to its large multi-photon cross-section which allows initiation of multiphoton absorption at low laser power thresholds, thereby reducing self-focusing of the laser beam and other non-linear optical effects (Applegate et al., 2015). As such, voids could be formed in within these gels nearly 1 cm below the gel surface, a significant enhancement over other materials tested (Oujja et al., 2009; Sarig-Nadir et al., 2009). These concepts were similarly applied in the context of preformed cellular aggregates of mesenchymal stem cells embedded in synthetic PEG-based matrices, into which perfusion channels were formed by ablating pulses of a picosecond infrared laser (Brandenberg and Lutolf, 2016). This technique has also been applied to other materials such as collagen type I, where perfused HUVECs within channels adhered to the walls of the newly created channels and formed a confluent EC layer. The idea of generating 3D structures within the bulk of a transparent cell-supporting hydrogel can be extended to selected other materials, with the clarity of the gels permitting the creation of patterns with features down to the micron scale and as deep as 1 cm within soft silk protein hydrogels (Applegate et al., 2015). In order to take full advantage of this method, the hydrogel material must have an appropriate multi-photon cross-section corresponding to the laser wavelength used for fabrication, which substantially limits the choice of available hydrogels. These material restrictions can be relaxed by introducing highly light-absorbing molecules into the fabrication volume. Indeed, when laser ablation of channels in a collagen scaffold was performed in the presence of fluorescein as a photo-absorption agent the laser exposure times were dramatically reduced and scanning velocities as high as 350–400 mm/s could be achieved, in a fluorescein concentration-dependent manner (Skylar-Scott et al., 2016). The obtained channels were perfused in a microfluidic device and their inner surface could be colonized by ECs. Remarkably, the diameter of the channels was approximately 50 μm, and there are seemingly no technical limitations to further reducing this dimension. Apart from thermal ablation, laser-induced photolysis has been suggested to generate tubular voids in hydrogels in presence of cells (Kloxin et al., 2010; Tibbitt et al., 2010), with one example using focused laser irradiation to induce localized dissociation of a PEG-based hydrogel (Tibbitt et al., 2010). While current studies have emphasized the potential of these materials for localized modulation of adhesion properties, these materials would likely be ideal substrates for on-demand free form generation of perfusion networks.

## Pro-angiogenic Matrix Engineering for Organoid and Vascular Cells

For endothelial cells to be induced to migrate and form *de novo* vessels in conjunction with organoid development there is a need for an additional element in a constructed angiogenesis-promoting microenvironment: a three-dimensional extracellular matrix which can be co-permissive and supportive for both angiogenesis as well as organoid growth. This implies that angiogenesis must occur in a matrix used to support the growth of specific organoids. In almost all current protocols, the extracellular matrix which supports three-dimensional organoid development is Matrigel, a one-size-fits-all hydrogel whose components are highly uncontrolled and whose properties cannot be readily manipulated (Kleinman and Martin, 2005). The advent of highly modular and controllable artificial extracellular matrices has enabled the possibility to precisely tailor materials to specific biological applications (Langer and Tirrell, 2004; Griffith and Swartz, 2006; Lutolf et al., 2009). Such matrices are now being translated into the organoid field (Meinhardt et al., 2014), not only to enhance the reproducibility of organoid generation, but also allow for a better understanding of role of the biophysical cues in 3D morphogenesis (Gjorevski et al., 2016; Cruz-Acuña et al., 2017). For example, neural tube organoids generated in optimized synthetic matrices have been shown to have more homogeneous phenotypes than those in Matrigel, as well as enhanced morphogenetic features such as higher frequency of apico-basal and dorso-ventral patterning (Ranga et al., 2016). In addition to the tremendous variety of material compositions which can be tested, the combinations of soluble factors which provide critical inductive cues which drive differentiation and morphogenesis can also be varied. 3D artificial ECM microarrays, which allow for a wide variety of combinatorial conditions to be assayed simultaneously, can be used to quickly and systematically investigate the role of these matrix and soluble factor combinations (Ranga et al., 2014b). Combined with powerful image analysis, data visualization and statistical analysis tools, such approaches can help in rapidly generating a regulatory landscape of fate and morphogenesis (Ranga et al., 2016). While a number of matrices have been developed specifically to induce angiogenesis, including by the incorporation of increasingly sophisticated VEGF patterns and guidance cues (Ehrbar et al., 2008; Sacchi et al., 2014), it remains to be ascertained whether such matrices are also compatible with tissue-specific organoid growth, and an ongoing challenge will remain to develop matrices amenable to increasingly specialized co-cultures. Alternatively, it is possible that multi-matrix composites will be required to compartmentalize co-cultures.

## Induced Angiogenesis in Engineered Tissues

The approaches described so far possess clear advantages such as full control over vascular network topology and immediate functionality. However, being predetermined by the fabrication process, such vascular structures cannot respond to dynamic changes in the environment, such as changes in oxygen consumption, tissue patterning, and growth. Thus, the presence of mechanisms of active vascular remodeling is a critical element of any tissue development model system. In addition to a direct fabrication of capillary networks or chemically guided cell patterning, novel approaches have recently been proposed to spontaneously vascularize engineered tissues (Gage and Fisher, 1991; Watson et al., 2014; Clevers, 2016; Pham et al., 2018) (Figure 1F). For example, the successful spontaneous vascularization of human lung fibroblast spheroids grown in microfluidic chip (Nashimoto et al., 2017) was recently demonstrated in a chip with three parallel fluidic channels separated by micro-posts allowing cell migration and proliferation between the channels (Figure 1F). Based on an earlier study standardizing spheroid cultures (Kunz-Schughart et al., 2006), human lung fibroblasts (hLF) and HUVECs were co-cultured to generate composite spheroids of approximately 600 μm in diameter. With the middle channel of the microfluidic chip seeded with spheroids and the adjacent side channels with HUVECs, the endothelial cells formed a network around and within which the spheroid and a fully perfusable vasculature was established. Interestingly, the hLFs and the HUVECs in the spheroid co-culture were shown to play different roles, with hLFs acting as a signaling source to stimulate network outgrowth toward the spheroid, and HUVECs promoting anastomosis of the sprouts from the side channels to the central spheroid-containing channel. Moreover, the most active form of angiogenesis was observed without exogenous VEGF present in medium, suggesting that interactions between hLFs and HUVECs were sufficient to drive the co-culture. This recent study is expected to be one of many which suggest that *in vitro*, as *in vivo*, interactions between vasculature and host/target tissue are bidirectional, with inductive roles for both.

## Organoid Vascularization via *in vivo* Organoid Transplantation

Despite recent advances with microfluidic or organ-on-chip approaches, fully functional organoid vascularization has thus far only been demonstrated by transplanting organoids into host animals, where native vasculature was seen to integrate into the ectopic implant. In most cases, initial *in vitro* co-culture with endothelial cells was required for vascularization and engraftment. In a first demonstration, human pluripotent stem cell (hiPSC)-derived liver buds were transplanted into immunodeficient mice (Takebe et al., 2013), with resulting formation of vascularized functional liver tissue capable of producing albumin starting from day 10 and up to day 45 post-transplantation. Remarkably, transplanted hIPSC-derived hepatocytes produced much less albumin, underscoring the importance of three-dimensional and vascularized tissue formation for functional use. Indeed, engraftment of immature organoids in vasculature-rich tissues such as kidney, lung or brain has been shown to result in invasion of the organoid by the host vascular network. Recently, a method for transplanting human brain organoids into the adult mouse brain has been developed (Mansour et al., 2018). Such organoid grafts progressively differentiated and matured, resulting in functional neuronal networks interconnected synaptically with host neuronal circuits, as demonstrated by extracellular recordings and optogenetic stimulation. Importantly, *in vivo* imaging revealed extensive infiltration of the host vasculature within a few days after transplantation.

## Strategies for *de novo* Design of *in vitro* Vasculature

The *in vivo* vascularization of organoids clearly demonstrates that, given the right conditions and timing, organoids can be perfused throughout. It is therefore important to ask: what is missing in approaches focused on direct vascularization? One major limitation in most currently generated *in vitro* vascular constructs is that reported dimensions, ranging from 150–200 μm (Miller et al., 2012) to 1–2 mm (Huber et al., 2016), do not correspond to physiological length scales required, as the typical diameter of a capillary is about 10 μm. Indeed, the regulation of blood flow to achieve an oxygen supply matched to oxygen demand is controlled at the level of the local microcirculation (Pittman, 2013) and the balance in local oxygen pressure gradients results from interactions of local diffusion and consumption demands. As such, it is critical to generate the right dimensional structures at that scale, and it is here that additive micromanufacturing such as stereolithography based on two-photon polymerization may be particularly suitable to enable the level of 3D resolution required. Despite the fact that direct fabrication by photopolymerization is a maturing technology, some practical fabrication aspects remain to be resolved. For example, further development of biocompatible hydrogel materials composition will be required to limit swelling and deformation of printed structures in aqueous media after fabrication (Huber et al., 2016). Similar issues arise with photo-induced removal of material, where a gel block is subjected to a focused laser light. Since thicker samples must be processed to generate larger vascular networks, objectives with long working distance are required, which normally have low-numerical aperture and thus lead to low spatial resolutions. For both photo-ablation and photo-degradation techniques another limitation which will have to be overcome in order to increase throughput is the high power and/or long illumination times needed, rendering the technique less practical. It is possible that printing small-diameter capillary networks can be avoided altogether, and that organoid vascularization can be achieved instead by guiding angiogenesis from larger vessels to anastomose toward growing organoids. This idea has thus far been limited by the lack of easy and reliable connection to an *in vitro* perfusion system, however recent vascularization-on-chip work (Nashimoto et al., 2017) demonstrates that such systems may be optimized to achieve flow through an organoid. Notably, the successful proliferation of such vascular networks and connection to an organoid tissue still requires co-culture of target organoid cells with VEGF-secreting cells in order to provide the necessary chemo-attractive angiogenic cues, and it remains to be seen whether such neo-tissues can produce these signals independently.

## Revisiting Design Principles for *de novo* Design of *in vitro* Vasculature

Beyond the technical aspects of generating vasculature, one important question relates to the design of the network itself. With printing technologies at various scales now allowing for unprecedented versatility in creating three-dimensional structures, it is necessary to consider the network geometries relevant for organoid development. Indeed, the physical interaction between blood flow, vascular wall structure and branching architecture have all been shown to play an important role in optimizing and regulating the function of vascular systems, and the design principles that govern the organization of such systems have been extensively investigated both at the theoretical and experimental levels.

One of the first and most enduring theoretical frameworks has been Murray's law, which state that the laws governing branching in vascular networks are based on the optimization of the energy required to maintain blood volume and velocity. An important consequence of Murray's law is that the tangential shear stress at the wall remains constant throughout the vascular network, for symmetric bifurcating systems (Barber and Emerson, 2010). Murray's law has been experimentally validated for large arteries and arterioles, however, it does not provide a good description of the microcirculation. Additional features have been added to this framework over the years, notably adding the energy costs associated with maintaining smooth muscle contraction against the distending effects of wall shear stress, improving on the rheological representation of blood as well as including the geometric and material complexities of the vessel walls (Alarcón et al., 2005). These improved theoretical models have been applied to simulations of various types of microfluidic networks. For example, a generalized version of Murray's law was derived for the design of microfluidic manifolds and hierarchical fluid distribution systems (Emerson et al., 2006). More recently, these theoretical insights have been applied, in simulations and in practice, on applications-oriented microfluidic networks. For instance, simulation-driven procedures for optimal design of cell-seeded scaffolds have been proposed based on combining oxygen transport with design principles governing vascular trees (Kang et al., 2013), with experimentally validated results pointing to the role of branching points in ensuring optimal diffusion. Simulation tools have also been used to model increasingly complex 3D branched networks encompassing multiple length scales, with hemodynamic effects of design parameters calculated throughout the tree structure. Such simulations allow for systematic investigations of variations in geometrical parameters such as vessel diameter, vessel length, bifurcation angle and fractal dimensions of the tree, and their role in hemodynamics (Yang and Wang, 2013). Alternatively, data-driven models have focused on relating measured diffusion gradients of oxygen in *in vitro* cultured tissues to spatial distributions of cell number and viability (Radisic et al., 2005). These empirical models could then be used to conclude that oxygen concentration and cell viability decreased linearly with distance from the perfused surface, while live cell density decreased exponentially, with physiological levels only present within a thickness of approximately 130 μm. Interestingly, quantitative and analytical solutions to diffusion equations for different tissue geometries with a particular focus on organoids (McMurtrey, 2016) have shown that oxygen diffusion, rather than nutrient transport is generally the primary limiting factor at steady state in metabolically active constructs, and that cell density could be tuned such that enhanced growth could be achieved if metabolically active and proliferative cells were positioned closer to the source of oxygen.

## Generating Biomimetic Vasculature Based on Developmental Principles

While studies investigating the design principles of vascular beds have searched for overarching descriptions and mechanisms for explaining observed mature *in vivo* networks, these may need to be revised in the context of a dynamically developing system. In particular, iPSC-derived organoids recapitulate, at least in part, key steps of embryonic cell fate specification, patterning, morphogenesis, and growth. In this context, it becomes clear that the dynamic interplay between a growing, diversifying tissue and its interpenetrating vascular network could be a critical missing element in ensuring that development occurs in a temporally prescribed manner. Therefore, an important issue which has been overlooked in the emergent field of organoid vascularization is the question of appropriate design of the vascular network not only in space but also in time. While printing at various scales is now possible it remains to be determined what is the optimal vascular structure to print at a specific developmental time point, leading to the development of an optimized overall printing strategy. One approach could be based on principles of biomimetic *in vitro* generation of developing, anatomical, site-specific vasculature. Indeed, the formation of blood vessels during embryonic development is a highly choreographed process where bidirectional interactions between the developing tissue and its developing vasculature allow for stereotypical patterning of the vascular tree (Walls et al., 2008). The *de novo* formation of blood vessels is initiated by the assembly of precursor cells, a process known as vasculogenesis, followed by the sprouting of new vessels from pre-existing ones, termed angiogenesis. In humans, fetal vascularization is initiated early in the third week of gestation (approximately day 16), concurrently with axis formation and organogenesis; for comparison, these events occur at day 7 in the mouse. Vasculogenesis begins by the formation of blood islands from hemangioblasts of mesodermal origin, which then differentiate into two cell populations: angioblasts (vascular precursors), which form endothelial cells, and hemocytoblasts, which form blood cell precursors. Angioblasts migrate and coalesce into cords to form primitive lumens, thereby establishing the first fluidic networks. Growth factors such as vascular endothelial growth factor (VEGF) and placental growth factor (PlGF) secreted by adjacent tissues are critical in stimulating the growth and development of such primordial vascular networks. Vasculogenesis is the dominant process of vessel formation in early embryogenesis, from which the major large arteries such as the dorsal aorta are formed. As primitive plexi are established, smaller vessels are generated by angiogenesis. Sprouting from pre-existing vessels leads to mature vascularization of multiple organs such as the brain. Importantly, the vascular tree undergoes constant remodeling during embryonic development, driven both by genetically encoded information as well as by hemodynamic forces (Walls et al., 2008). While the earliest endothelial cells are molecularly distinguished by markers such as endoglin, von-Willebranf factor, CD31, VE-cadherin, Tie2, EphB4 and EphrinB2 (Augustin and Koh, 2017) and are not specified to a particular organ, vascular function becomes increasingly specialized in parallel with increasing organ specification. This molecular and cellular specialization is critical to achieve the required organ-specific function. For example, the endothelial cells in continuous capillaries are bound by tight junctions which ensure that most large molecules, drugs and pathogens do not cross the blood-brain barrier, whereas those in the sinusoidal capillaries of organs such as the liver, where freer exchange of large solutes is required, present completely different morphologies and large gaps. The early steps of pan-endothelial differentiation are generally well-understood, however the role of specific inductive cues for later specification and differentiation has only more recently been explored. Recent work analyzing and comparing transcriptomes, accessible chromatin, and DNA methylome signatures from mouse brain, liver, lung, and kidney endothelial cells (Sabbagh et al., 2018) is beginning to reveal the gene regulatory networks that control such specification, which is expected to lead to improved protocols for *in vitro* derivation of more organ-specific cells. The development of vasculature clearly does not involve endothelial cells alone: other cell types such as pericytes, smooth muscle cells and immune cells play varied roles in providing structural and functional support as well as signaling guidance, and will also be required to build more complete *in vitro* models. While current strategies of co-culture of endothelial cells with organoids are an important starting point, thus far, there has been no evidence of spontaneous vascularization in organoid systems (i.e., without exogenous addition of endothelial/support cells), most likely due to the fact that the vast majority of organoid models involve directed differentiation to specific organ systems, which presumably precludes the formation of alternative fates such as endothelium. Indeed adding the staged endothelial and support cells at the developmentally correct time point and in the appropriate geometrical relationship to the developing organoid will likely prove to be both necessary and highly challenging. The advent of increasingly detailed *in vivo* single cell transcriptomic and image-based spatial information (e.g., from lattice light-sheet microscopy) from early development to adult tissues provides a detailed roadmap for recreating these tissues *in vitro*, and will be particularly valuable for PSC-derived systems.

## Conclusion

Today the advent of increasingly accurate and scalable 3D microfabrication technologies allows for unprecedented possibilities in generating free-form vascular structures. The deployment of these technologies in the context of organoid culture could help overcome the lack of vascularization in these model systems, which remains one of the major challenges in the field. Optimal approaches will involve finding a balance between the explicit structural patterning imposed by the printing process and the self-organizing nature of the organoid and the vascular network surrounding and interpenetrating it. We have shown in this review that constructing gel-based architectures can take many forms, with each being most appropriate depending on the desired dimensional and temporal scale. Depending on the methods used, the interaction between vasculature and organoid can be engineered to occur simultaneously or sequentially, and the spatial relationship can be designed to incorporate multi-material compartments and dimensions. Bioprinting in the form of filament or droplet deposition offers rapid layer-by-layer construction possibilities at the mesoscale (circa 1 mm) with a versatile material toolbox. Conversely, DMD and 2-photon fabrication offer unprecedented precision at the micro-scale, with limitations mainly in throughput and requirement for the use of photopolymerizable materials. With all such additive manufacturing options, a significant limitation is that the constructed structures are not readily modifiable after their initial creation, with, for example, the initial printing of a static vasculature followed by organoid seeding. A unique advantage of subtractive methods is that vessels or other structural guiding elements can be created at arbitrary points in time, conforming to organoid growth and development. While sacrificial networks must first be built up using additive manufacturing techniques, laser ablation allows for more flexibility in determining arbitrary structural configurations after organoid seeding. Laser ablation is limited however to the horizontal plane as the possibility to carve out space within a gel presupposes a clear optical path and precludes the creation of structures directly above or below the organoids. Additionally, these methods can only be used with polymers which have specific absorbing properties with the used wavelength, and local heat buildup may be unsuitable in regions close to organoids. Ultimately, leveraging the power of single or combinations of these 3D biofabrication technologies to create truly biomimetic, vascularized organoids will require integrating molecular and structural lessons from the embryonic development of both the target tissue as well as that of its specific microvasculature. Indeed, technological tools such as the ones we have described will allow for highly spatially and temporally defined interactions between organoids and their supporting vasculature, which will allow the exploration of minimal conditions for self-organization and larger-scale growth and maturation. Microvascular patterning and organ-on-a-chip microfluidic technology will increasingly allow for the incorporation of the right cells at the right place, such that perfusable organoids can serve as ever-more reliable model systems for basic and translational applications.

## Author Contributions

All authors have contributed to writing, editing and revising of the manuscript, and have approved it for publication.

### Conflict of Interest Statement

The authors declare that the research was conducted in the absence of any commercial or financial relationships that could be construed as a potential conflict of interest.
